# A Pharmacological Examination of the Cardiovascular Effects of Malayan Krait (*Bungarus candidus*) Venoms

**DOI:** 10.3390/toxins9040122

**Published:** 2017-03-29

**Authors:** Janeyuth Chaisakul, Muhamad Rusdi Ahmad Rusmili, Wayne C. Hodgson, Panadda Hatthachote, Kijja Suwan, Anjaree Inchan, Lawan Chanhome, Iekhsan Othman, Krongkarn Chootip

**Affiliations:** 1 Department of Pharmacology, Phramongkutklao College of Medicine, Bangkok 10400, Thailand; 2 Kulliyyah of Pharmacy, International Islamic University Malaysia, Bandar Indera Mahkota, Kuantan 25200, Malaysia; rusdirusmili@iium.edu.my; 3 Monash Venom Group, Department of Pharmacology, Biomedical Discovery Institute, Monash University, Clayton, VIC 3800, Australia; 4 Department of Physiology, Phramongkutklao College of Medicine, Bangkok 10400, Thailand; hatthachoteting@gmail.com (P.H.); kijja_suwan@yahoo.com (K.S.); 5 Department of Physiology, Faculty of Medical Science, Naresuan University, Phitsanulok 65000, Thailand; anjaree.in@gmail.com (A.I.); krongkarnc@gmail.com (K.C.); 6 Queen Saovabha Memorial Institute, The Thai Red Cross Society, Bangkok 10330, Thailand; lchanhome@yahoo.com; 7 Jeffrey Cheah School of Medicine and Health Sciences, Monash University Sunway Campus, Bandar Sunway 46150, Malaysia; iekhsan.othman@monash.edu

**Keywords:** venom, Malayan krait, cardiovascular, rat, hypotension

## Abstract

Cardiovascular effects (e.g., tachycardia, hypo- and/or hypertension) are often clinical outcomes of snake envenoming. Malayan krait (*Bungarus candidus*) envenoming has been reported to cause cardiovascular effects that may be related to abnormalities in parasympathetic activity. However, the exact mechanism for this effect has yet to be determined. In the present study, we investigated the in vivo and in vitro cardiovascular effects of *B. candidus* venoms from Southern (BC-S) and Northeastern (BC-NE) Thailand. SDS-PAGE analysis of venoms showed some differences in the protein profile of the venoms. *B. candidus* venoms (50 µg/kg–100 µg/kg, i.v.) caused dose-dependent hypotension in anaesthetised rats. The highest dose caused sudden hypotension (phase I) followed by a return of mean arterial pressure to baseline levels and a decrease in heart rate with transient hypertension (phase II) prior to a small decrease in blood pressure (phase III). Prior administration of monovalent antivenom significantly attenuated the hypotension induced by venoms (100 µg/kg, i.v.). The sudden hypotensive effect of BC-NE venom was abolished by prior administration of hexamethonium (10 mg/kg, i.v.) or atropine (5 mg/kg, i.v.). BC-S and BC-NE venoms (0.1 µg/kg–100 µg/mL) induced concentration-dependent relaxation (EC_50_ = 8 ± 1 and 13 ± 3 µg/mL, respectively) in endothelium-intact aorta. The concentration–response curves were markedly shifted to the right by pre-incubation with L-NAME (0.2 mM), or removal of the endothelium, suggesting that endothelium-derived nitric oxide (NO) is likely to be responsible for venom-induced aortic relaxation. Our data indicate that the cardiovascular effects caused by *B. candidus* venoms may be due to a combination of vascular mediators (i.e., NO) and autonomic adaptation via nicotinic and muscarinic acetylcholine receptors.

## 1. Introduction

Envenoming by kraits (Genus *Bungarus*) is common in South Asia and some regions of Southeast Asia [1,2,3]. There are three species of krait found in Thailand, Indonesia and Malaysia, namely, *Bungarus candidus* (Malayan krait), *Bungarus fasciatus* (banded krait) and *Bungarus flaviceps* (red-headed krait) [4]. In Thailand, the Malayan krait is a category 1 medically important venomous snake, a category for species causing high levels of mobility and mortality [5]. The Malayan krait is characterized by a cylindrical body with 25–36 black cross-bands separated by white interspaces [4].

Clinically, neurotoxicity is the most significant manifestation following Malayan krait envenoming, which has been attributed to the presence of pre- and post-synaptic neurotoxins in the venom [6,7]. Interestingly, symptoms which are not related to neuromuscular blockade such as hyponatremia, rhabdomyolysis, and cardiovascular disturbances including hypertension and shock have been reported in envenomed patients in Vietnam [2].

Cardiovascular disturbances following snake bite are a life-threatening phenomenon leading to morbidity and mortality in victims bitten by vipers [8] and elapids [9]. Venom-induced cardiac arrest was reported to be caused by the venom prothrombin activator, causing intravenous coagulation [10]. However, our previous studies have shown that elapid phospholipase A_2_ (PLA_2_) may also be responsible for cardiovascular effects causing a sudden hypotensive effect via the release of dilator autacoids and direct vascular smooth muscle relaxation [11,12].

Severe hypertension was found to be a significant outcome following Vietnamese *B. candidus* envenoming where 33.3% of envenomed patients displayed systolic blood pressure exceeding 150 mmHg on two or more occasions [2]. This outcome was postulated to be due to elapid envenoming-induced autonomic dysfunction which could be due to neurotoxin blockade at presynaptic α_2_- adrenoceptors, causing an increase in catecholamine release [13]. Autonomic dysfunction has been reported following Malayan krait envenoming in Thailand where victims displayed a decrease in parasympathetic activities as indicated by mydriasis, hypertension, constipation and tachycardia [14].

Krait venoms contain a wide range of proteins and peptides which may contribute to cardiovascular dysfunction including natriuretic peptides, snake venom metalloproteinases (SVMP) and PLA_2_s [7]. In addition, components of snake venoms such as bradykinin potentiating peptides, L-type Ca^2+^ channel blockers and natriuretic peptides may contribute to cardiovascular dysfunction following envenoming [15].

Although cardiovascular disturbances seem to be a significant manifestation observed in Malayan krait envenomed patients, the mechanisms behind these effects have not been fully investigated. Further understanding of the pathology of krait envenoming-induced cardiovascular disturbances would have significant benefit in improving the management of severe krait envenoming (e.g., guiding early first aid or encouraging a focus on cardiovascular monitoring).

The aim of the current study was to determine the physiological changes in cardiovascular function following the administration of Malayan krait (*B. candidus*) venoms from two different geographical locations (i.e., Northeastern and Southern Thailand) in an anaesthetised rat model. We also studied the effect of Malayan krait venom on vascular function in isolated rat aorta preparations.

## 2. Results

### 2.1. Sodium Dodecyl Sulphate–Polyacrylamide Gel Electrophoresis (SDS–PAGE)

**Figure 1:**
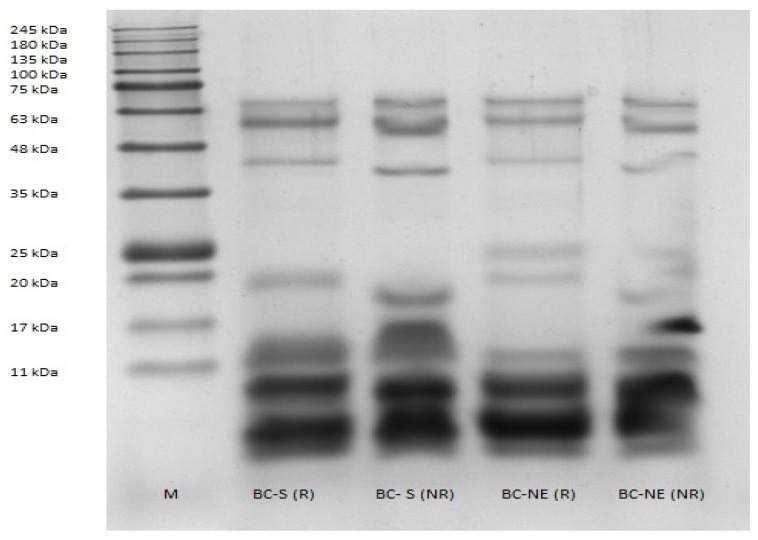
Sodium Dodecyl Sulphate–Polyacrylamide Gel Electrophoresis (SDS–PAGE) of venoms on a 10% separating gel with 5% stacking gel. Venoms were treated in reducing or non-reducing buffer prior to loading, electrophoresis, and stained with Coomassie Blue. M indicates the protein marker lane, BC-S indicates *B. candidus* venom from Southern Thailand and BC-NE indicates *B. candidus* venom from Northeastern Thailand. (R) indicates venom treated with reducing sample buffer and (NR) indicates venom treated with non-reducing sample buffer.

The venoms of *B. candidus* from Southern (BC-S) and Northeastern (BC-NE) Thailand were resolved in a gel under reducing and non-reducing conditions. SDS–PAGE analysis of venoms shows that there were differences in intensity and presence of protein bands (Figure 1). BC-NE venom possessed a greater number of protein bands compared to BC-S venom. Thick and high intensity bands clumped together were observed in the MW range below 17 kDa in reduced and non-reduced BC-S venoms. High intensity protein bands of BC-NE venom were detected at a MW < 11 kDa, in reducing and non-reducing buffers. No protein band was observed within the range of 25 kDa–35 kDa in reduced and non-reduced BC-S venoms. At a MW of 25 kDa, reduced BC-NE venom showed an obvious protein band while non-reduced BC-NE venom displayed an incomplete separation of protein bands in the MW range of 17 kDa–25 kDa.

### 2.2. Anaesthetised Rats

#### 2.2.1. Hypotensive Effect of *B. candidus* Venoms

**Figure 2:**
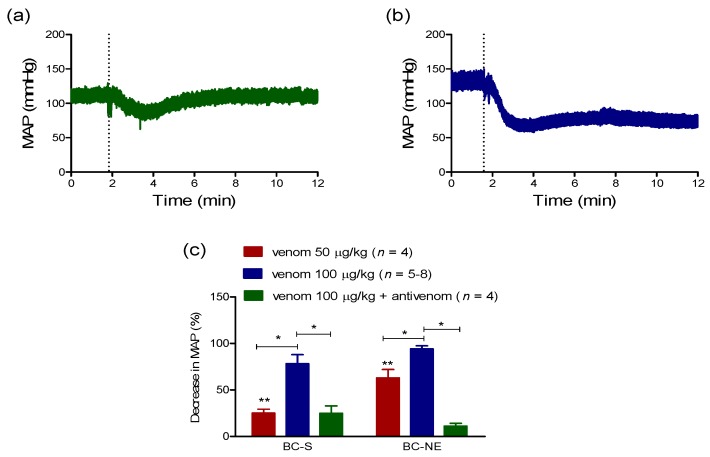
Traces showing the effect of (**a**) BC-S and (**b**) BC-NE venoms (50 µg/kg, i.v.) on MAP of anaesthetised rats. (**c**) Decrease in MAP following the administration of BC-S or BC-NE venom (50–100 µg/kg, i.v.) in the presence or absence of monovalent *B. candidus* antivenom at the recommended titer (i.e., 1 mL per 0.4 mg of venom). * *p* < 0.05, significantly different from venom 100 µg/kg (i.v.), Student’s unpaired *t*-test. ** *p* < 0.05, significantly different between groups, Student’s unpaired *t*-test.

*B. candidus* venoms (BC-S and BC-NE) produced a marked hypotensive effect in anaesthetised rats. BC-S and BC-NE venoms (50 µg/kg, i.v., Figure 2a,b) reduced mean arterial pressure (MAP) by 25 ± 4% and 63 ± 9%, respectively (*n* = 4, Figure 2c) while a larger dose of BC-S and BC-NE venoms (100 µg/kg, i.v., *n* = 5–8, Figure 2c) caused 87 ± 5% and 94 ± 3% reductions in MAP, respectively. Prior administration of monovalent *B. candidus* antivenom (i.e., 1 mL per 0.4 mg of *B. candidus* venom) significantly attenuated the hypotensive effect of BC-S (*n* = 4) and BC-NE (*n* = 4) venoms (100 µg/kg, i.v., Figure 2c).

#### 2.2.2. The Recovery in MAP Following Hypotensive Effect of *B. candidus* Venoms

**Figure 3:**
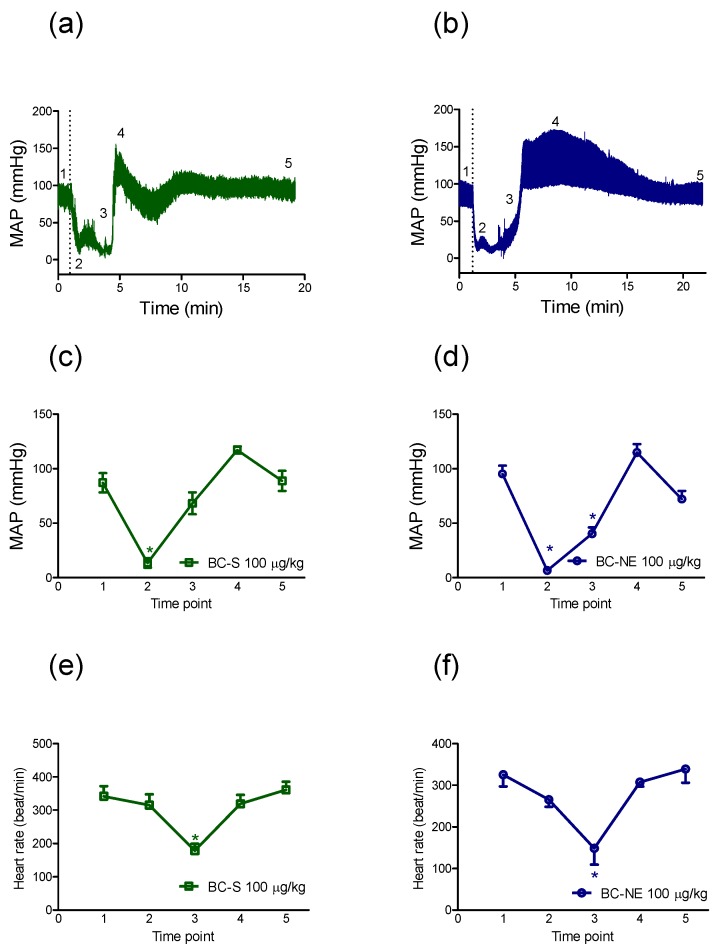
Traces of (**a**) BC-S (100 µg/kg, i.v.) and (**b**) BC-NE venoms (100 µg/kg, i.v.) on MAP in an anaesthetised rat at time point 1 (before venom injection), 2 (10 s after venom injection), 3 (50% recovery of MAP), 4 (the peak increase in MAP) and 5 (plateau in MAP, 20 min after venom injection). Effects of (**c**) BC-S venom (100 µg/kg, i.v., *n* = 4) and (**d**) BC-NE venom (100 µg/kg, i.v., *n* = 4) on MAP in different rats at time points 1, 2, 3, 4 and 5. Effects of (**e**) BC-S venom (100 µg/kg, i.v., *n* = 4) and (**f**) BC-NE venom (100 µg/kg, i.v., *n* = 4) on heart rate in different rats at time points 1, 2, 3, 4 and 5. * *p* < 0.05, significantly different from time point 1 (before venom injection), Student’s paired *t*-tests.

Both *B. candidus* venoms (BC-S and BC-NE, 100 µg/kg, i.v.) caused marked circulatory disturbances (Figure 3a, b) as characterized by a sudden decrease in MAP, when administered to anaesthetised rats (at time point 2), followed by a slight recovery in MAP (at time point 3) with a transient hypertensive effect (at time point 4) being observed. Further instability of MAP was not observed for at least 20 min following venom administration (at time point 5). However, a recovery of cardiovascular function was not observed in 25% of animals treated (complete cardiac collapse) by BC-NE venom (100 µg/kg, i.v., *n* = 2).

Administration of BC-S venom (100 µg/kg, i.v., Figure 3c) reduced MAP from 87 ± 9 mmHg to 13 ± 4 mmHg (*n* = 4) without a significant change in heart rate at time point 2 (i.e., 342 ± 30 bpm to 315 ± 32 bpm, Figure 3e). A slight recovery in MAP was recorded 3–4 min after venom administration with a significant decrease in heart rate (i.e., 178 ± 20 bpm, *p* < 0.05, Student’s paired *t*-test) at time point 3 until a hypertensive effect was observed, however this increase in MAP was not significant.

The recovery in MAP following the sudden decrease in MAP was also observed following the administration of BC-NE (100 µg/kg, i.v., Figure 3d) where MAP was significantly decreased from 95 ± 7 mmHg to 6 ± 1 mmHg (*n* = 6, *p* < 0.05, Student’s paired *t*-test). A recovery in cardiac function was recorded until MAP reached 114 ± 7 mmHg, then MAP decreased to 72 ± 7 mmHg (Figure 3d) 20 min after BC-NE venom administration. A significant decrease in heart rate following the administration of BC-NE venom (100 µg/kg, i.v., Figure 3f) was also recorded at time point 3 (i.e., from 325 ± 27 bpm at time point 1 to 148 ± 39 bpm at time point 3, *p* < 0.05, Student’s paired *t*-test).

#### 2.2.3. Effect of BC-NE Venom on MAP in the Presence of Receptor Antagonists

**Figure 4:**
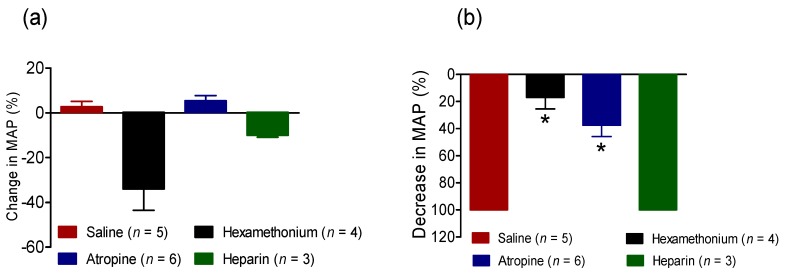
(**a**) Change in MAP of anaesthetised rats following the administration of saline (*n* = 5), hexamethonium (10 mg/kg, i.v., *n* = 4), atropine (5 mg/kg, i.v., *n* = 6) or heparin (300 units/kg, i.v., *n* = 3); (**b**) Effect of *B. candidus* venom from Northeastern Thailand (BC-NE; 100 µg/kg, i.v.) in the absence and presence of hexamethonium, atropine and heparin. * *p* < 0.05, is significantly different from saline, one-way ANOVA.

Prior administration of atropine (5 mg/kg, i.v., *n* = 6) or hexamethonium (10 mg/kg, i.v., *n* = 4), significantly attenuated the rapid hypotensive response induced by subsequent administration of BC-NE venom (100 µg/kg, i.v.) compared to vehicle control (saline, Figure 4b, *n* = 5). In contrast, the hypotensive effect of BC-NE (100 µg/kg, i.v.) was not significantly attenuated by prior administration of heparin (300 units/kg, i.v., *n* = 3) (*p* < 0.05, one-way ANOVA, Figure 4b).

### 2.3. Effect of B. candidus Venoms on Rat Aortic Rings

**Figure 5:**
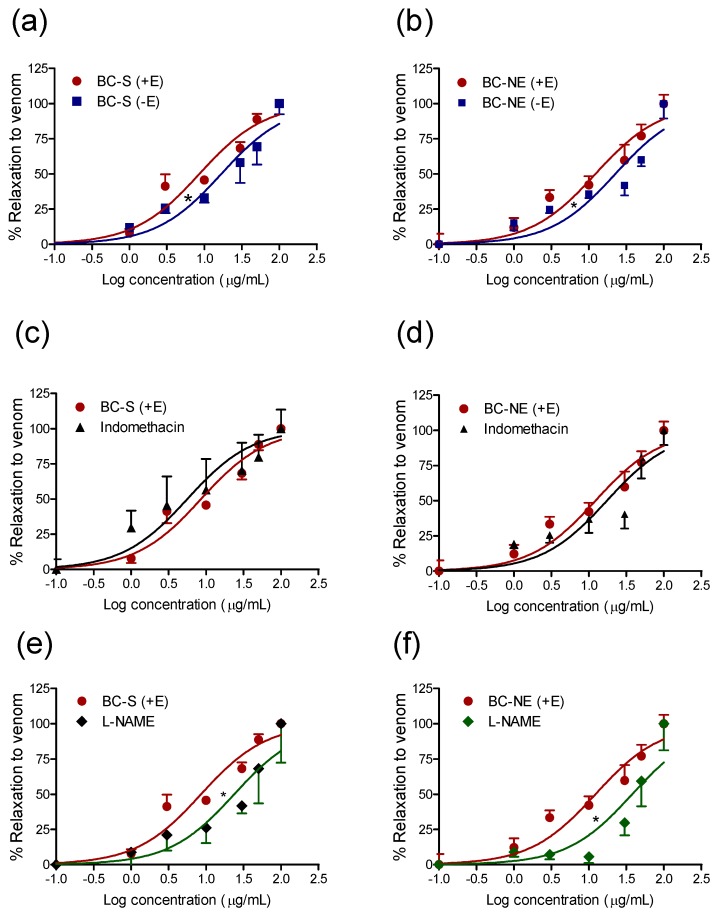
Concentration–response curves to (**a**) BC-S and (**b**) BC-NE venoms (0.1 µg/mL–100 µg/mL) in endothelium-intact and endothelium-denuded rat aortic rings (*n* = 4). Relaxation effect of (**c**) BC-S and (**d**) BC-NE venoms on endothelium-intact rat aortic rings in the presence and absence of indomethacin (10 µM, *n* = 5). Relaxation effect of (**e**) BC-S and (**f**) BC-NE venoms on endothelium-intact rat aortic rings in the presence and absence of L-NAME (0.2 mM, *n* = 5). * *p* < 0.05, is significantly different, Student’s unpaired *t*-test.

The effects of *B. candidus* venoms were determined on isolated phenylephrine pre-contracted rat aorta. BC-S and BC-NE venoms (0.1 µg/mL–100 µg/mL, *n* = 4) induced concentration-dependent relaxations in endothelium-intact aorta (EC_50_ = 8 ± 1 and 13 ± 3 µg/mL, respectively). In endothelium-denuded arteries, the concentration–relaxation curve was significantly shifted to the right in the presence of BC-S (Figure 5a, EC_50_ = 19 ± 4 µg/mL, *n* = 4) or BC-NE (Figure 5b, EC_50_ = 22 ± 3 µg/mL, *n* = 4) venoms.

In addition, a significant rightward shift of the concentration relaxation curves in endothelium-intact aorta was observed when L-NAME (0.2 mM) was added prior to the addition of BC-S (Figure 5e, EC_50_ = 20 ± 10 µg/mL, *n* = 5) or BC-NE (Figure 5f, EC_50_ = 32 ± 11 µg/mL, *n* = 5) venoms. However, indomethacin (10 µM) did not cause a significant rightward shift of the concentration–relaxation curve to either BC-S (Figure 5c, *n* = 5) or BC-NE (Figure 5d, *n* = 5) venom in endothelium-intact aorta. Both venoms (0.1 µg/mL–100 µg/mL) failed to induce aortic contraction in endothelium-intact aorta (n = 3, data not shown).

## 3. Discussion

Malayan krait (*Bungarus candidus*) is an elapid species found in Southeast Asia. The venom contains highly potent neurotoxins that inhibit neurotransmission at the neuromuscular junction [6]. Interestingly, symptoms involving cardiovascular function such as tachycardia and blood pressure irregularities which are not related to the neuromuscular blocking activity of the venom have been reported in some victims [2]. So far, the potential mechanisms behind these cardiovascular events have yet to be identified.

Previous studies have shown that animal venom composition is associated to season, habitat, prey type, inter-and intra-species including geographical variation [16,17]. These variations can be clinically significant as they can produce different outcomes following envenoming e.g., differences in cytotoxicity and cardiovascular effects [17]. In the present study, the protein band profiles of Malayan krait venoms from Northeastern and Southern Thailand were determined using SDS–PAGE analysis. In the reducing buffer, both venoms displayed less protein bands compared to non-reduced venoms indicating the presence of high amounts of protein complexes in both venoms. There were also notable differences between the venoms in the presence and intensity of protein bands at 25 kDa, suggesting variation in venom composition that could be due to geographical differences. In reducing and non-reducing buffers, both venoms showed thick and high protein bands in the MW range below 17 kDa which would likely be due to presynaptic PLA_2_ and three-finger neurotoxins [18].

In our experiments, the effect of geographical variation of Malayan krait venoms on hypotensive effect was significant, observed in animals receiving the lower (50 µg/kg, i.v.) but not the higher (100 µg/kg, i.v.) dose of venom. The mechanisms behind Malayan krait venom-induced hypotension have been postulated to include Ca^2+^ channel blocker activity [19] and the presence of natriuretic peptides [7,20]. Indeed, the proteomic profile of *B. candidus* venom indicated the presence of a natriuretic peptide but not Ca^2+^ channel blocker [7]. Moreover, autonomic dysfunction due to a blockade of adrenoceptors by elapid neurotoxins has been suggested to contribute to the hypertension observed following snake envenoming [13]. Interestingly, both episodes of hypertension or hypotension associated with shock have been observed following krait envenoming in a similar number of patients (33.3% and 31%, respectively) [2].

In the present study, BC-S and BC-NE venoms (50 µg/kg–100 µg/kg, i.v.) produced a dose-dependent hypotensive effect. However, venoms did not significantly alter heart rate as indicated by a comparison of heart rate immediately prior to venom injection and then again at the time point where MAP had decreased by 80%. Administration of venom at the higher dose (i.e., 100 µg/kg, i.v.) caused a triphasic effect which was characterized by an immediate decrease in blood pressure (phase I), followed by a transient hypertension (phase II) and then a return to basal levels with a slight reduction in MAP (phase III). The return of MAP following envenoming is similar to the effect of snake venom PLA_2_ [12] and giant jellyfish *Nemopilema nomurai* venom [21]. Moreover, complete cardiovascular collapse was observed in some animals administered BC-NE, but not BC-S, venom (100 µg/kg, i.v.).

In the current study, a significant bradycardia was observed at time point 3 where MAP had recovered by approximately 50%. However, we did not observe tachycardia which is different from a previous clinical report in which three victims displayed an increase in heart rate [14]. This might be due to differences in the response of different species [22] or an effect of anesthetic which can blunt reflex responses [23].

Heparin has been postulated to inhibit histamine release from canine mast cells [24]. In a previous study, prior administration of heparin protected rats from Papuan taipan (*Oxyuranus scutellatus*) venom-induced cardiovascular collapse, suggesting the involvement of anaphylactic mediator release [11]. In the current study, heparin did not inhibit *B. candidus* venom-induced hypotension, suggesting also that the release of histamine is not associated with this response. However, the effects of Malayan krait venom were markedly attenuated by pre-treatment with hexamethonium or atropine, indicating the involvement of ganglionic nicotinic and autonomic muscarinic receptors, respectively.

Antivenom is the only reliable treatment for systemically envenomed patients [25]. The Queen Saovabha Memorial Institute (Thai Red Cross Society, Bangkok, Thailand) is a manufacturer of antivenoms for medically important Southeast Asian snake species. Administration of *B. candidus* antivenom prevented hypotension from Malayan krait venom in anaesthetised rats. This indicates that the toxins that induce hypotension in the venoms are antigenically homologous.

In vascular experiments, the venoms caused concentration-dependent relaxation in both endothelium-intact and endothelium-denuded rat aortae. Relaxation curves were shifted to the right when the tissues were pre-incubated with the nitric oxide (NO) synthase inhibitor, L-NAME or removal of the endothelium, suggesting that the endothelium-dependent relaxant effect is mediated by NO. However, this effect requires further investigation in different vascular beds. In our previous studies in rat mesenteric artery preparations, the relaxant effect of Papuan taipan venom and its purified PLA_2_ involved a combination of the release of dilator autacoids (i.e., PGI_2_) and a direct effect on vascular smooth muscle which was attenuated by indomethacin and the protein kinase A inhibitor, Rp-8-CPT-cAMP [11,12]. This venom-induced vasodilation may assist the elapid neurotoxins to reach their targets. The transient hypertensive response observed following sudden hypotension may not be due to a direct effect of venom on the vasculature as venom-induced contraction was not observed in aortae experiments. In addition, there was no significant difference in the relaxation caused by BC-S and BC-NE venoms, indicating that the venom components that cause relaxation are present in venom from snakes from both localities.

We have demonstrated that Thai *B. candidus* causes profound cardiovascular effects characterized by sudden hypotension, and activation of autonomic cardiovascular reflexes. Further purification and characterisation of the venom components responsible for the cardiovascular activities may enable the better management of Malayan krait envenoming.

## 4. Conclusions

These data indicate that the cardiovascular disturbance observed after envenoming by Malayan krait may involve autonomic reflex and vascular nitric oxide mechanisms. Early basic life support and monitoring of cardiovascular function may be required to prevent and manage the life-threatening outcomes.

## 5. Materials and Methods

### 5.1. Venom Preparation and Storage

Freeze-dried Malayan krait (*B. candidus*) venoms were obtained from Queen Saovabha Memorial Institute (QSMI) of the Thai Red Cross Society, Bangkok, Thailand. The venoms were milked and pooled from specimens collected in Nakhon Ratchasima, Northeastern Thailand (BC-NE) and Nakhon Si Thammarat, Southern Thailand (BC-S). The snake venoms from each region were milked by directly attaching a microhaematocrit tube on each fang. The collected venom was then transferred to a 1.5 mL microcentrifuge tube, frozen at −20 °C and freeze-dried. Freeze-dried venom samples were weighed, labeled and stored at −20 °C prior to use. When required, the venoms were weighed and dissolved in distilled water. Dissolved venoms were kept on ice during experiments.

### 5.2. Protein Concentration Determination

Venom protein content was determined using a BCA Protein Assay Kit (Pierce Biotechnology, Rockford, IL, USA) as per the manufacturer’s instructions. Briefly, 25 µL of protein was loaded onto a 96-well plate in triplicate, then 200 µL reagent buffer mix was added to each well. The plate was incubated at 37 °C for 30 min, then read at 562 nm using a plate reader spectrophotometer (Enspire multimode plate reader, Waltham, MA, USA). Protein concentration was determined from the standard curve.

### 5.3. Sodium Dodecyl Sulphate–Polyacrylamide Gel Electrophoresis (SDS–PAGE)

Venoms (12.5 μg) in reducing and non-reducing sample buffers were resolved and electrophoresed at 90 V in 10% separating gel with 5% stacking gel using the method previously described [26]. Protein bands were visualized by staining with Bio-Safe Coomassie G-250 solution (Bio-Rad Laboratories; Hercules, CA, USA), followed by de-staining using distilled water. BLUelf Prestained Protein Ladder (GeneDirex, Taiwan) was electrophoresed in the gel as protein molecular weight marker. The gel was scanned using the Fusion FX (Vilber Lourmat, Collegien, France).

### 5.4. Anaesthetised Rat Preparation

Male Sprague-Dawley rats weighting 280 g–330 g were anaesthetised with pentobarbital sodium (50 mg/kg–70 mg/kg, i.p.). Additional anaesthetic was administered throughout the experiment as required. A midline incision was made in the cervical region, and cannulae were inserted into the trachea, jugular vein and carotid artery, for artificial respiration (if required) and administration of drugs/venom and measurement of blood pressure, respectively. Arterial blood pressure was recorded using a Gould Statham P23 pressure transducer filled with heparinised saline (25 U/mL). Systemic blood pressure was monitored on a MacLab system (ADInstruments). At the conclusion of the experiment, the animals were killed by an overdose of pentobarbitone (i.v.). Pulse pressure was defined as the difference between systolic and diastolic blood pressures. Mean arterial pressure (MAP) was defined as diastolic blood pressure plus one-third of pulse pressure. The rats were kept under a heat lamp during the experiment.

Where indicated, hexamethonium bromide 10 mg/kg (i.v.) [27] or atropine 5 mg/kg (i.v.) [11] were administered to inhibit autonomic ganglionic and muscarinic receptors, respectively. Heparin (300 U/kg, i.v.) was administered to block the release of histamine [28]. Monovalent *B. candidus* antivenom (Lot No.: BC00115) at the recommended titer (i.e., 1 mL per 0.4 mg of *B. candidus* venom) was administered via the jugular vein (i.v. bolus). Control rats were injected with the same volume of normal saline (0.9% sodium chloride, i.v.). All drugs, saline and antivenom were given 15 min prior to venom administration.

### 5.5. Isolation and Study of Rat Aortic Ring

Male Wistar rats (200 g–250 g) were anaesthetised with sodium pentobarbital (50 mg/kg–70 mg/kg, i.p.), then the chest was cut open and the thoracic aorta removed and placed in ice-cold physiological saline solution (PSS) composed of (mM): NaCl 122; KCl 5; (N-(2-hydroxyethyl)piperazine N’-(2-ethanesulfonic acid)) (HEPES) 10; KH_2_PO_4_ 0.5; NaH_2_PO_4_ 0.5; MgCl_2_ 1; glucose 11; and CaCl_2_ 1.8, pH adjusted to 7.3 with NaOH. The aorta was cleared of surrounding loose connective tissue and fat, and cut into 2 mm–5 mm lengths. Where indicated, the endothelium was removed by gently rubbing the intimal surface with a thin stainless steel wire. Aortic rings were mounted on a pair of intraluminal wires in tissue chambers containing PSS as described previously [29]. Pre-heated PSS (3 mL) was added to the bath, bubbled with air and maintained at 37 **°**C. Tissue segments were allowed to equilibrate for 1 h at a resting tension of 1 g during which time the solution was changed every 15 min. An intact endothelium was confirmed by a maximal relaxation to 10 µM acetylcholine (ACh) in tissues precontracted with a sub-maximal concentration of phenylephrine (1 µM). Arteries that produced relaxations greater than 80% were considered to have an endothelium intact. Cumulative vasorelaxation responses to venom (0.1 µg/mL–100 µg/mL) were performed in both endothelium-intact and endothelium-denuded aortic rings. The isometric tension was measured using a force transducer (CB Sciences Inc., Milford, CT, USA) connected to a MacLab system (ADInstruments).

Where indicated, L-NAME (0.2 mM) and indomethacin (10 µM) [30] were used to inhibit NO production and prostaglandin production, respectively.

### 5.6. Chemical and Drugs

Monovalent *B. candidus* antivenom (Lot No.: BC00115) was purchased from Queen Saovabha Memorial Institute (QSMI) of the Thai Red Cross Society, Bangkok, Thailand. The following drugs and solutions were purchased from Sigma Aldrich (St. Louis, MO, USA): ACh, L-NAME, indomethacin, atropine and hexamethonium bromide. Heparin was obtained from LEO Pharma (Ballerup, Denmark).

### 5.7. Data Analysis and Statistics

Statistical analysis was performed using Prism 6.0 software (GraphPad Software, La Jolla, CA, USA). Student’s unpaired *t*-test performed on venom responses in the presence of venoms in different animals and tissues, and paired *t*-tests were used to compare responses before and after venom/agonist in the same animal or tissue. Multiple comparisons were made using a one-way analysis of variance (ANOVA) followed by Tukey’s multiple comparison. Values of *p* < 0.05 were accepted as significant. Data were expressed as mean ± SEM.

### 5.8. Animal Ethics

The rats were purchased from the National Laboratory Animal Centre, Mahidol University, Salaya, Nakhon Pathom, Thailand. The animals were housed with free access to food and drinking water. Approval for all experimental procedures was granted by the Subcommittee for Multidisciplinary Laboratory and Animal Usage, Phramongkutklao College of Medicine and the Animal Ethics Committee, Naresuan University (Permits for approved experiments: NU-AEE590506).

## References

[1] Silva A., Maduwage K., Sedgwick M., Pilapitiya S., Weerawansa P., Dahanayaka N.J., Buckley N.A., Johnston C., Siribaddana S., Isbister G.K. (2016). Neuromuscular effects of common krait (*Bungarus caeruleus*) envenoming in Sri Lanka. PLoS Negl. Trop. Dis..

[2] Trinh K.X., Khac Q.L., Trinh L.X., Warrell D.A. (2010). Hyponatraemia, rhabdomyolysis, alterations in blood pressure and persistent mydriasis in patients envenomed by Malayan kraits (*Bungarus candidus*) in southern Vietnam. Toxicon.

[3] Viravan C., Looareesuwan S., Kosakarn W., Wuthiekanun V., McCarthy C.J., Stimson A.F., Bunnag D., Harinasuta T., Warrell D.A. (1992). A national hospital-based survey of snakes responsible for bites in Thailand. Trans. R. Society. Trop. Med. Hyg..

[4] Chanhome L., Cox M.J., Vasaruchapong T., Chaiyabutr N., Sitprija V. (2011). Characterization of venomous snakes of Thailand. Asian Biomed..

[5] WHO (2016). Venomous snakes of the south-east asia region, their venoms and pathophysiology of human envenoming. Guidelines for the Management of Snake-Bites.

[6] Khow O., Chanhome L., Omori-Satoh T., Ogawa Y., Yanoshita R., Samejima Y., Kuch U., Mebs D., Sitprija V. (2003). Isolation, toxicity and amino terminal sequences of three major neurotoxins in the venom of Malayan krait (*Bungarus candidus*) from Thailand. J. Biochem..

[7] Rusmili M.R., Yee T.T., Mustafa M.R., Hodgson W.C., Othman I. (2014). Proteomic characterization and comparison of Malaysian *Bungarus candidus* and *Bungarus fasciatus* venoms. J. Proteom..

[8] Silva A., Pilapitiya S., Siribaddana S. (2012). Acute myocardial infarction following a possible direct intravenous bite of Russell's viper (*Daboia russelli*). BMC Res. Notes.

[9] Chaisakul J., Isbister G.K., Kuruppu S., Konstantakopoulos N., Hodgson W.C. (2013). An examination of cardiovascular collapse induced by eastern brown snake (*Pseudonaja textilis*) venom. Toxicol. Lett..

[10] Tibballs J., Sutherland S.K., Rivera R.A., Masci P.P. (1992). The cardiovascular and haematological effects of purified prothrombin activator from the common brown snake (*Pseudonaja textilis*) and their antagonism with heparin. Anaesth. Intensiv. Care.

[11] Chaisakul J., Isbister G.K., Konstantakopoulos N., Tare M., Parkington H.C., Hodgson W.C. (2012). *In vivo* and *in vitro* cardiovascular effects of Papuan taipan (*Oxyuranus scutellatus*) venom: Exploring "sudden collapse". Toxicol. Lett..

[12] Chaisakul J., Isbister G.K., Tare M., Parkington H.C., Hodgson W.C. (2014). Hypotensive and vascular relaxant effects of phospholipase A2 toxins from Papuan taipan (*Oxyuranus scutellatus*) venom. Eur. J. Pharmacol..

[13] Agarwal R., Aggarwal A.N., Gupta D. (2006). Elapid snakebite as a cause of severe hypertension. J. Emerg. Med..

[14] Laothong C., Sitprija V. (2001). Decreased parasympathetic activities in Malayan krait (*Bungarus candidus*) envenoming. Toxicon.

[15] Joseph R., Pahari S., Hodgson W.C., Kini R.M. (2004). Hypotensive agents from snake venoms. Current. Drug Targets. Cardiovasc. Haematol. Disord..

[16] Chippaux J.P., Williams V., White J. (1991). Snake venom variability: Methods of study, results and interpretation. Toxicon.

[17] Winter K.L., Isbister G.K., McGowan S., Konstantakopoulos N., Seymour J.E., Hodgson W.C. (2010). A pharmacological and biochemical examination of the geographical variation of *Chironex fleckeri* venom. Toxicol. Lett..

[18] Rusmili M.R., Yee T.T., Mustafa M.R., Othman I., Hodgson W.C. (2014). In-vitro neurotoxicity of two Malaysian krait species (*Bungarus candidus* and *Bungarus fasciatus*) venoms: Neutralization by monovalent and polyvalent antivenoms from Thailand. Toxins.

[19] Chanhome L., Sitprija V., Chaiyabutr N. (2010). Effect of *Bungarus candidus* (Malayan krait) venom on general circulation and renal hemodynamics in experimental animals. Asian Biomed..

[20] Siang A.S., Doley R., Vonk F.J., Kini R.M. (2010). Transcriptomic analysis of the venom gland of the red-headed krait (*Bungarus flaviceps*) using expressed sequence tags. BMC Mol. Biol..

[21] Kim E., Lee S., Kim J.S., Yoon W.D., Lim D., Hart A.J., Hodgson W.C. (2006). Cardiovascular effects of *Nemopilema nomurai* (scyphozoa: Rhizostomeae) jellyfish venom in rats. Toxicol. Lett..

[22] Hart A.J., Isbister G.K., O'Donnell P., Williamson N.A., Hodgson W.C. (2013). Species differences in the neuromuscular activity of post-synaptic neurotoxins from two Australian black snakes (*Pseudechis porphyriacus* and *Pseudechis colletti*). Toxicol. Lett..

[23] Hanamoto H., Niwa H., Sugimura M., Morimoto Y. (2012). Autonomic and cardiovascular effects of pentobarbital anesthesia during trigeminal stimulation in cats. Int. J. Oral Sci..

[24] Inase N., Schreck R.E., Lazarus S.C. (1993). Heparin inhibits histamine release from canine mast cells. Am. J. Physiol..

[25] Johnston C.I., Ryan N.M., O’Leary M.A., Brown S.G., Isbister G.K. (2017). Australian taipan (*Oxyuranus* spp.) envenoming: Clinical effects and potential benefits of early antivenom therapy - Australian snakebite project (asp-25). Clin. Toxicol. (Phila).

[26] Laemmli U.K. (1970). Cleavage of structural proteins during the assembly of the head of bacteriophage t4. Nature.

[27] Ismail A., Mohamed M., Sulaiman S.A., Wan Ahmad W.A. (2013). Autonomic nervous system mediates the hypotensive effects of aqueous and residual methanolic extracts of *Syzygium polyanthum* (wight) walp. Var. Polyanthum leaves in anaesthetized rats. Evidence.-Based Complement. Altern. Med.: ECAM.

[28] Tibballs J., Sutherland S.K. (1992). The efficacy of heparin in the treatment of common brown snake (*Pseudonaja textilis*) envenomation. Anaesth. Intensiv. Care.

[29] Kamkaew N., Scholfield C.N., Ingkaninan K., Maneesai P., Parkington H.C., Tare M., Chootip K. (2011). *Bacopa monnieri* and its constituents is hypotensive in anaesthetized rats and vasodilator in various artery types. J. Ethnopharmacol..

[30] Crachi M.T., Hammer L.W., Hodgson W.C. (1999). A pharmacological examination of venom from the Papuan taipan (*Oxyuranus scutellatus canni*). Toxicon.

